# Modeling of Xerostomia After Radiotherapy for Head and Neck Cancer: A Registry Study

**DOI:** 10.3389/fonc.2020.01647

**Published:** 2020-08-14

**Authors:** Eva Onjukka, Claes Mercke, Einar Björgvinsson, Anna Embring, Anders Berglund, Gabriella Alexandersson von Döbeln, Signe Friesland, Giovanna Gagliardi, Clara Lenneby Helleday, Helena Sjödin, Ingmar Lax

**Affiliations:** ^1^Medical Radiation Physics and Nuclear Medicine, Karolinska University Hospital, Stockholm, Sweden; ^2^Department of Oncology-Pathology, Karolinska Institute, Stockholm, Sweden; ^3^Department of Oncology, Karolinska University Hospital, Stockholm, Sweden; ^4^Epistat Epidemiology and Statistics Consulting, Uppsala, Sweden

**Keywords:** xerostomia, head and neck cancer, cox regression, nomogram, registry analysis

## Abstract

**Aim:**

Data from a local quality registry are used to model the risk of late xerostomia after radiotherapy for head and neck cancer (HNC), based on dosimetric- and clinical variables. Strengths and weaknesses of using quality registry data are explored.

**Methods:**

HNC patients treated with radiotherapy at the Karolinska University hospital are entered into a quality registry at routine follow up, recording morbidity according to a modified RTOG/LENT-SOMA scale. Other recorded parameters are performance status, age, gender, tumor location, tumor stage, smoking status, chemotherapy and radiotherapy data, including prescribed dose and organ-at-risk (OAR) dose. Most patients are entered at several time points, but at variable times after treatment. Xerostomia was modeled based on follow-up data from January 2014 to October 2018, resulting in 753 patients. Two endpoints were considered: maximum grade ≥2 (XER_G≥2_) or grade ≥3 (XER_G≥3_) late xerostomia. Univariate Cox regression was used to select variables for two multivariate models for each endpoint, one based on the mean dose to the total parotid volume (D_tot_) and one based on the mean dose to the contralateral parotid (D_contra_). Cox regression allows the estimation of the risk of xerostomia at different time points; models were presented visually as nomograms estimating the risk at 9, 12, and 24 months respectively.

**Results:**

The toxicity rates were 366/753 (49%) for XER_G≥2_ and 40/753 (5.3%) for XER_G≥3_. The multivariate models included several variables for XER_G≥2_, and dose, concomitant chemotherapy and age were included for XER_G≥3_. Induction chemotherapy and an increased number of fractions per week were associated with a lower risk of XER_G≥2_. However, since the causality of these relationships have limited support from previous studies, alternative models without these variables were also presented. The models based on the mean dose to the total parotid volume and the contralateral parotid alone were very similar.

**Conclusion:**

Late xerostomia after radiotherapy can be modeled with reasonable predictive power based on registry data; models are presented for different endpoints highly relevant in clinical practice. However, the risk of modeling indirect relationships, given the unavoidably heterogeneous registry data, needs to be carefully considered in the interpretation of the results.

## Introduction

Radiotherapy contributes to favorable control of disease in the great majority of patients with head and neck cancer (HNC) and will certainly remain one of the prominent integral components in the multidisciplinary management of this disease. Approximately 80% of the patients receive radiotherapy at least once during the course of their disease (1). Even with great advances in radiotherapy planning facilitating a tailored delivery of radiation dose, some damage will be inflicted on normal cells in tissues adjacent to the tumor. The treatment of HNC is associated with clinically significant radiation-induced toxicity, especially in combination with concurrent systemic agents, chemotherapy or biomodulators (2, 3).

One of the most frequently reported side effects is hyposalivation, and subsequent xerostomia, due to co-irradiation of the salivary glands. Chronic xerostomia is a multifactorial process which can affect quality of life profoundly. The process includes reduced salivary output, decreased salivary pH and increased viscosity of the saliva (4, 5). This may result in the unpleasant sensation of dry mouth, altered taste, dysfunction of mastication, swallowing dysfunction and difficulties with speech (6–8). Xerostomia is also associated with changes in the normal flora of the mouth, which increases the risk of oral infections including dental caries (9). The reported prevalence of xerostomia in the normal population ranges from 5.5 to 46% (10). It increases with age, partly due to the frequent use of multiple medications by the elderly (11, 12). In a retrospective study including over 12,000 dental patients, predictors for patient-reported xerostomia were intake of more medications, recreational drug use, rheumatic diseases, psychiatric diseases, eating disorders and radiotherapy (10). Thus, the risk of xerostomia in patients undergoing radiotherapy for HNC is dependent on clinical factors as well as the radiation dose.

The incidence of HNC is currently on the rise as more patients suffer from an HPV-related tumor, predominantly in the oropharynx. These patients have a good prognosis with respect to tumor control and will have to live a long life with potential treatment-related side effects. It is therefore of utmost importance to clarify further the dose/volume-response relationship, also for lower grades of radiation-induced xerostomia. The introduction of new, more conformal, techniques such as intensity-modulated radiotherapy (IMRT) has reduced the rate of xerostomia both with respect to measurement of saliva flow and quality of life. There is a consensus that xerostomia is sufficiently limited by keeping the mean dose to the total parotid volume below 26 Gy as a planning criterion (13). However, as this criterion is frequently violated in order to achieve adequate tumor coverage, the rate of xerostomia in HNC patients is still a concern (14). Also, the relative importance of each parotid gland for preserved salivary function is unclear (15).

Evidence-based radiotherapy requires models which can be used for treatment planning, based on representative datasets. It has been recognized that such models need to be continuously validated and updated (16, 17) as treatment protocols and patient populations evolve (16). For this purpose, registries of outcome data need to be implemented, maintained and analyzed; the limited size of datasets historically used for model fitting, and the homogeneous nature of the data provided by controlled clinical trials, put into question the applicability of these models as decision-support tools in clinical practice. Also, one or a few fixed follow-up times are considered, not considering the risk of toxicity as a function of time. In the current study, Cox regression, where the endpoint is time to event, is used and thus the risk of xerostomia at any time point after treatment can be estimated. To the best of our knowledge, this is the first model of this type developed for xerostomia.

In this registry study, a multivariate model of xerostomia is fitted using clinical-, patient-, and treatment-related parameters in an effort to better predict both more severe but also moderate grades of xerostomia in the individual patient. The real-world nature of the collected data, and the size of the dataset, are favorable for the application of the model as a decision-support tool when treating HNC patients. The risk of modeling indirect relationships, given the unavoidably heterogeneous registry data, is carefully considered in the choice of model, variable selection method and interpretation of the results.

## Materials and Methods

### Quality Registry

Head and neck cancer patients who are treated with radiotherapy at the Karolinska University Hospital, either radically or postoperatively with or without chemotherapy, are followed up after their therapy according to local protocols every third to fourth month during the first 2 years and thereafter every 6 months for an additional 3 years. Since 2013, clinical follow-up data, as well as patient- and treatment-related parameters are entered into a quality-registry database for all patients. A modified Radiation Therapy Oncology Group (RTOG)/LENT-SOMA scale is used for skin-, mucosa-, larynx- and mandible toxicity as well as xerostomia, dysphagia and trismus. Morbidity appearing within 3 months of completed radiotherapy is categorized as acute while morbidity appearing later is categorized as late. Performance status evaluated according to WHO/ECOG/ZUBROD is also registered at every follow-up visit. Patient-related parameters collected are: gender, age, tumor location, tumor stage, HPV association (for patients with cancer in the oropharynx) and smoking status at the time of treatment. The treatment parameters collected are: data on induction chemotherapy, concomitant drug therapy as well as prescribed dose and organ-at-risk (OAR) dose.

Informed consent regarding participation in the quality registry, including in the publication of analyzed data, is obtained from each patient. The study was approved by the regional ethics committee (2016/268-31/1).

### Patients

The patients included in this study had histologically confirmed HNC originating in and categorized as cancer of the oral cavity, oropharynx or other (epipharynx, hypopharynx, nasal cavity, paranasal sinuses or metastases in the neck with an unknown primary). Patients with a tumor location associated with very low parotid dose were excluded (larynx, lip and basalioma) and tumors in the parotid were excluded since these patients can only be considered to have one single parotid as OAR.

None of the included patients had previous head and neck radiotherapy or previous malignancy except non-melanoma skin cancer. Radiotherapy was prescribed curatively, alone or with induction chemotherapy and/or concomitant cisplatin or cetuximab. Cisplatin was prescribed according to the estimated surface area of the patient as follows: 40 mg/m^2^ (maximum 70 mg) once weekly, or 80 mg/m^2^ (maximum 160 mg) every third week, for the duration of the radiotherapy course. Cetuximab was prescribed with the first dose (400 mg/m^2^, maximum 800 mg) one week before the start of radiotherapy and thereafter a weekly dose of 250 mg/m^2^ (maximum 500 mg) for the duration of the radiotherapy course, i.e., typically six additional doses. Follow-up data in this study are from January 2014 to October 2018, relating to radiotherapy mainly from 2010 to May 2018, though a few records in the registry relate to even earlier treatments. The compliance in the registration of follow-up data for patients in this study was about 70%, resulting in 753 patients.

Xerostomia was assessed by oncologists specialized in treating HNC. The assessment was based both on visual inspection of the oral cavity and on the description of symptoms described by the patient. The endpoints of this study were late xerostomia of grade 2–4 (moderately dry, completely dry and fibrosis) and grade 3 to 4 late xerostomia (completely dry and fibrosis), respectively; the maximum grade registered for each patient was considered. The two endpoints will hereafter be referred to as XER_G≥2_ and XER_G≥3_.

Baseline (before radiotherapy) xerostomia scores were not available in this study. Thus, all endpoints reflect the overall xerostomia status after treatment, not exclusively relating to the treatment.

### Treatment

External-beam radiotherapy was delivered with a linear accelerator using 6 MV photons. Twenty-five percent of the patients were treated with a combination of external-beam and brachy radiotherapy. The majority of the external-beam treatments were delivered with IMRT but 7% received 3D-conformal radiotherapy. During treatment planning the parotid glands and larynx were considered the primary OAR followed by swallowing structures; target coverage had the highest priority. Target volumes for primary tumor and regional nodal groups at risk of harboring occult metastatic disease, as well as OAR, were delineated according to departmental guidelines, which also include dose constraints. Specifically, the parotids were delineated as the entire gland as visible in the CT images, including both the deep lobe and the superficial lobe.

Prescribed dose to the primary target volume was; >73 Gy in 12%, 68 Gy in 69%, 66 Gy in 8%, and 50 Gy in 9%. The dose per fraction was 2.2 Gy for the highest dose group and 2.0 Gy for the other groups. Fifty-one percent of the patients were treated with six fractions per week and 45% received five fractions per week. The dose per fraction to elective volumes was 2.0 Gy or 1.52 Gy when treating with a sequential- and simultaneous-integrated boost, respectively.

In this study, the mean dose to ipsilateral- and contralateral parotid glands separately, as well as the mean dose for the two glands together was considered, from the external-beam radiotherapy only. A separate investigation, made on a limited number of patients, showed that the contribution to the parotid mean dose from brachytherapy was negligible for modeling purposes (95%-percentile: 1.5 Gy).

### Modeling

Since the time to the registered score varied from patient to patient, multivariate Cox regression models were developed. For each endpoint, the time to event was defined as the interval between the end of radiotherapy and the first score exceeding the respective threshold. Hazard ratios and nomograms were produced for each model. A bootstrap validation with 1000 samples was performed and the mean C-statistic over the bootstrap samples was used as a measure of model performance. Calibration was performed using bootstrap cross validation with 100 bootstrap samples, as described in (18), using Harrell’s R packages. All analyses were performed in R.

The candidate explanatory variables were: mean dose to the total parotid volume (D_tot_), mean dose to the contralateral parotid (D_contra_), mean dose to the ipsilateral parotid (D_ipsi_), number of treatment fractions per week, tumor location, T stage, N stage, smoking status, induction chemotherapy, concomitant chemotherapy, gender and age. Two alternative models, based on D_tot_ and D_contra_, respectively, were considered. This dose variable was forced into the model, irrespective of the univariate significance for the endpoint, and D_ipsi_ was only a candidate variable for the D_contra_ model, to avoid direct dependence between variables. For each model, the candidate variables with *p* ≤ 0.2 in a univariate analysis were considered in the multivariate analysis, while an alpha value of 0.05 was used in the multivariate analysis. Categorical variables were considered significant if at least half of the groups passed the alpha value.

Given the lack of register-data analyses available for comparison, and that only internal validation was possible, an alternative selection of variables was made following the univariate analysis, excluding any variables with no/limited support in the literature with regard to their contribution to the risk of xerostomia. These variables were induction chemotherapy and the number of treatment fractions per week.

## Results

### Patients

Out of the 753 patients included in the analysis, there were 366 (49%) with grade ≥ 2 late xerostomia and 40 (5.3%) with grade ≥3 late xerostomia. The median follow-up was 363 days and 292 days for XER_G≥2_ and XER_G≥3_ respectively. Table 1 lists the descriptive statistics.

**TABLE 1:** Patient- and treatment characteristics.

	Patients	%
**Mean parotid dose**		
D_tot_ (Gy); mean (SD)	26.2 (8.2)	
D_contra_ (Gy); mean (SD)	17.7 (7.2)	
D_ipsi_ (Gy); mean (SD)	34.8 (13)	
**Fractions per week**		
5	294	39
6	437	58
10	22	2.9
Age (years); mean (SD)	61.4 (11.4)	
**Gender**		
Female	249	33
Male	504	67
**Tumor location**		
Oral cavity	172	23
Oropharynx	443	59
Other	138	18
**T stage**		
0	40	5.3
1	174	23
2	289	38
3	108	14
4	142	19
**N stage**		
0	252	34
1	85	11
2a/b	348	46
2c	61	8.1
3	7	0.9
**Smoking**		
No/never	299	40
Smoker	152	20
Previous	302	40
**Concomitant chemotherapy**		
Cisplatin	254	34
ERBIT	206	27
No	293	39
**Induction chemotherapy**		
Yes	203	27
No	550	73

### Univariate Analysis

The results from the univariate analysis (hazard ratios and *p*-values) are listed in Supplementary Table A1 in the Supplementary Material, where the variables considered in the multivariate analysis are highlighted in bold. The main dose variable (D_tot_ or D_contra_) did not consistently obtain low *p*-values but according to the chosen model selection strategy, this variable was still included in the multivariate model. More variables were significant for grade ≥2 xerostomia than for ≥3 xerostomia. This might be due to the fewer events of high-grade xerostomia in the population.

Radiotherapy technique (3D-conformal vs. IMRT) was not considered as an explanatory variable since any effect would be indirect, through cross-correlation with the volume of exposed parotid. However, it’s association with xerostomia was nonetheless tested in the univariate analysis. Surprisingly, as seen in Supplementary Table A1, the risk of xerostomia was significantly reduced in the small group of patients with 3D-conformal radiotherapy; these patients were typically treated for unilateral targets and received a lower target dose, as well as a lower D_tot_.

### Multivariate Analysis

**FIGURE 1:**
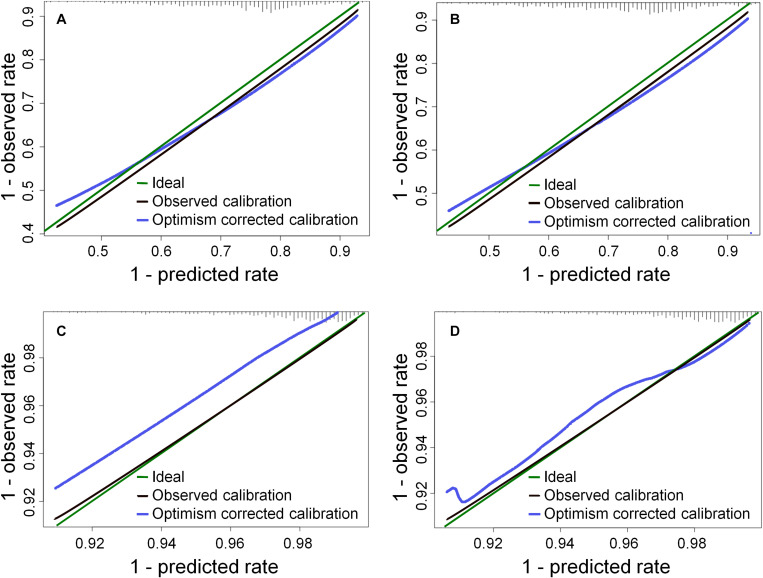
Calibration plots for the models in Table 2 at 1 year: **(A)** the D_tot_ model for XER_G≥2_, **(B)** the D_contra_ model for XER_G≥2_, **(C)** the D_tot_ model for XER_G≥3_, **(D)** the D_contra_ model for XER_G≥3_. The histogram on the upper × axis represents the frequency distribution of 1 minus the predicted probabilities (c.f. a survival analysis).

Two multivariate models were fitted to each endpoint, one based on D_tot_ and one on D_contra_; in a second step the models were refitted to only the variables associated with *p* < 0.05 in the initial multivariate model – see Table 2. The models are well calibrated – see calibration plots in Figure 1.

**TABLE 2:** Hazard ratios and corresponding *p*-values (in brackets) for the Cox regression multivariate analysis.

Endpoint	Included variables	Hazard ratio (*p*-value)	
		Model: D_tot_	Model: D_contra_
XER_G≥2_	Mean parotid dose (D_tot_ or D_contra_)	0.98 (0.033)	1.004 (0.65)
	D_ipsi_	–	0.99 (<0.01)
	Fractions per week; reference = 5		
	6	1.02 (0.86)	0.997 (0.99)
	10	0.33 (<0.01)	0.32 (<0.01)
	Tumor location; reference = oral cavity		
	Oropharynx	1.12 (0.50)	1.14 (0.44)
	Other	0.60 (<0.01)	0.62 (0.016)
	N stage; reference = 2c		
	0	0.45 (<0.01)	0.50 (<0.01)
	1	0.43 (<0.01)	0.49 (<0.01)
	2a/b	0.59 (<0.01)	0.65 (0.017)
	3	0.24 (0.018)	0.27 (0.031)
	Concomitant chemotherapy; reference = no		
	Cisplatin	1.73 (<0.01)	1.66 (<0.01)
	Erbitux	1.37 (0.50)	1.32 (0.088)
	Induction chemotherapy; reference = no		
	Yes	0.62 (<0.01)	0.62 (<0.01)
	Age	1.01 (0.023)	1.011 (0.023)
	**C-statistic (SE)**	**0.64 (0.057)**	**0.64 (0.057)**
XER_G≥3_	Mean parotid dose (D_tot_ or D_contra_)	1.01 (0.054)	1.02 (0.26)
	Concomitant chemotherapy; reference = no		
	Cisplatin	2.57 (0.012)	2.46 (0.017)
	Erbitux	0.96 (0.94)	0.92 (0.87)
	Age	1.06 (<0.01)	1.06 (<0.01)
	**C-statistic (SE)**	**0.67 (0.017)**	**0.68 (0.017)**

*The xerostomia endpoints were grade ≥ 2 (XER_G≥2_) and grade ≥ 3 (XER_G≥3_). The bootstrap-validation C-statistic is listed below the included variables.*

XER_G≥3_ showed a positive dependence on dose, as opposed to XER_G≥2_, and the performance of this model was better. The dose variable was not statistically significant in all models but many non-dosimetric variables were significant, especially in the models for XER_G≥2_. The models based on D_tot_ and D_contra_ were very similar.

**FIGURE 2:**
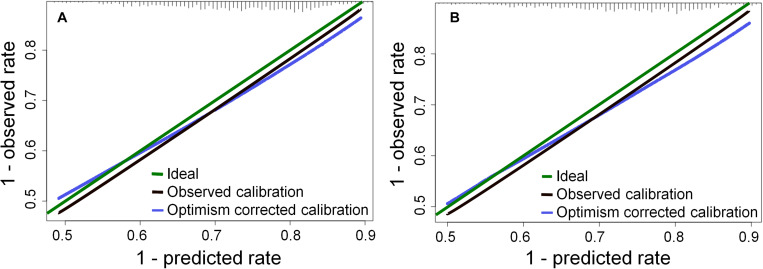
Calibration plots for the models in Table 3 at 1 year: **(A)** the D_tot_ model for XER_G≥2_, **(B)** the D_contra_ model for XER_G≥2_, The histogram on the upper × axis represents the frequency distribution of 1 minus the predicted probabilities (c.f. a survival analysis).

Given the explorative nature of the above analysis, an alternative variable selection method was also applied for the multivariate analysis by excluding any non-consensus variables. While in Table 2 the models revealed a lower risk of XER_G≥2_ for patients with an accelerated treatment schedule and induction chemotherapy, the causality in these relationships might be controversial. Thus, the XER_G≥2_ models were refitted, excluding the number of fractions per week and induction chemotherapy – see Table 3. As two variables were excluded the performance of the models reduced somewhat. The calibration plots are shown in Figure 2.

**TABLE 3:** Hazard ratios and corresponding *p*-values (in brackets) for the Cox regression multivariate analysis excluding non-consensus variables for the XER_G≥2_ models.

Endpoint	Included variables	Hazard ratio (*p*-value)	
		Model: D_tot_	Model: D_contra_
XER_G≥2_	Mean parotid dose (D_tot_ or D_contra_)	0.98 (<0.01)	0.997 (0.68)
	D_ipsi_	–	0.99 (<0.01)
	Tumor location; reference = oral cavity		
	Oropharynx	1.07 (0.67)	1.07 (0.67)
	Other	0.57 (<0.01)	0.58 (<0.01)
	N stage; reference = 2c		
	0	0.58 (<0.01)	0.62 (0.020)
	1	0.57 (0.014)	0.61 (0.042)
	2a/b	0.69 (0.027)	0.73 (0.080)
	3	0.31 (0.052)	0.33 (0.067)
	Concomitant chemotherapy; reference = no		
	Cisplatin	2.02 (<0.01)	1.98 (<0.01)
	Erbitux	1.46 (0.011)	1.42 (0.019)
	Age	1.02 (<0.01)	1.02 (<0.01)
	**C-statistic (SE)**	**0.60 (0.057)**	**0.60 (0.057)**

*The bootstrap-validation C-statistic is listed below the included variables.*

**FIGURE 3:**
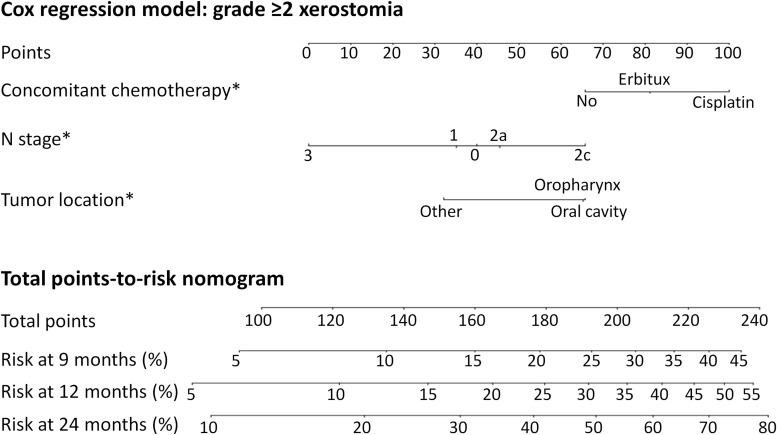
The predicted risk of grade ≥2 xerostomia at 9, 12, and 24 months after radiotherapy, not including dose, due to the inverse dose-response relationship. Model specifics are listed in Supplementary Table A2 in the Supplementary Material. *Note that the model does not imply significant differences between all categories with respect to the endpoint – the *p*-value only refers to the difference from the reference category (see Supplementary Table A2 for *p*-values). “No” was reference for concomitant chemotherapy and Erbitux was not significantly different from the reference. “N2c” was reference for N stage and N3 was not statistically different. Note that the model does not establish whether there is a difference between N0 and N1. “Oral cavity” was reference for tumor location and oropharynx was not statistically different.

**FIGURE 4:**
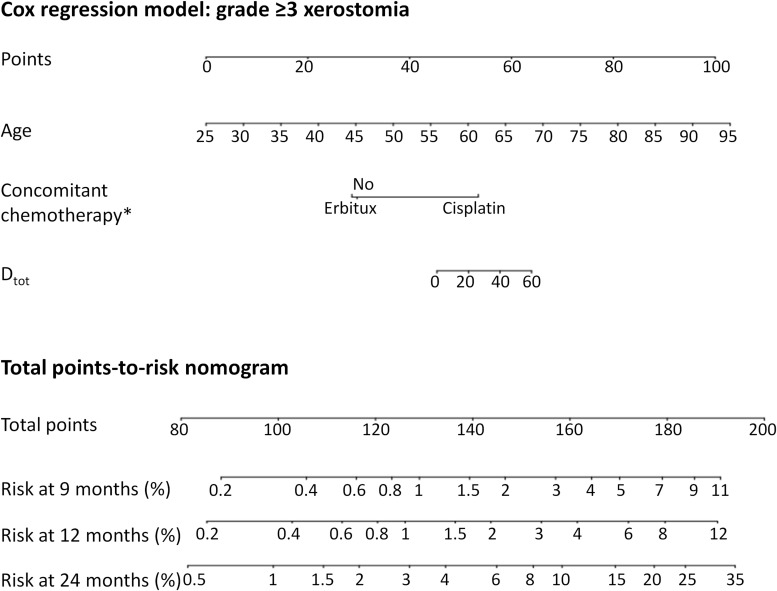
The predicted risk of grade ≥3 xerostomia at 9, 12, and 24 months after radiotherapy, using the D_tot_ dose variable. ^∗^Note that not all categories are significantly different from each other with respect to the endpoint – the *p*-value only refers to the difference from the reference category (see Table 2 for *p*-values). **“**No**”** was reference for concomitant chemotherapy and Erbitux was not significantly different.

Since the models for XER_G≥2_ had an inverse dose-response these are not suitable as decision support tools. Instead, Figure 3 shows a nomogram where dose was not forced into the model (for model specifics and the calibration plot, see Supplementary Table A2 and Supplementary Figure A1 in the Supplementary Material). Figure 4 shows the nomogram for XER_G≥3_ corresponding to the model listed in Table 2. Please refer to the Supplementary Material for an example of how to read the nomograms.

## Discussion

While the rate of xerostomia is reduced by sparing the parotid glands, a probable contributor to persistent xerostomia could be radiation-induced damage to other salivary glands such as the submandibular glands, sublingual glands and minor salivary glands in the oral cavity. Studies have shown a correlation between the dose to submandibular glands and sticky saliva (19). Some studies have also demonstrated that in selected patients it is safe to try to spare the submandibular glands with IMRT; however, this approach should be used with caution in patients with a significant risk of recurrence, relating to tumor location (20). The impact on xerostomia from minor salivary glands in the oral cavity and oropharynx is difficult to study because of their anatomical dispersion and their poor anatomical definition in the planning image. Data are conflicting regarding the benefit of reducing the dose to these glands (19, 21) but delineation of the oral cavity as an OAR with respect to xerostomia has been recommended in some institutions. In the present analysis, the only OAR considered was the parotid gland since other salivary glands had not been consistently delineated and the corresponding dose is not recorded in the registry. Also, the series of studies by Beetz et al. (19, 22, 23) suggest that the parotid is the main OAR for xerostomia (although using a slightly different definition compared to the present study), by showing that only the dose to the parotids, among the different salivary glands, was a significant predictor of patient-rated xerostomia.

The dose to the *contralateral* parotid has been studied as a predictor for patient-rated xerostomia (19) and salivary function is largely preserved if at least one parotid receives less than 25 Gy of mean dose (24). Still, there seems to be a benefit of limiting the dose to both parotids (25). Given the uncertainty of the definition of the OAR, both the total parotid volume and the contralateral parotid volume were considered, in different models. These models were very similar but applying the model for the contralateral parotid only, to optimize treatment plans, could be expected to lead to an increase in *ipsilateral* parotid dose. Such a strategy is not supported by the current study; all patient plans were optimized to spare both parotids as much as the target coverage allowed.

Baseline xerostomia is not uncommon in HNC patients and is predictive of xerostomia after radiotherapy (19, 26). Therefore, patients with baseline xerostomia are often excluded from studies of purely radiation-induced xerostomia. In contrast, the aim of the current study was to develop a model based on real-world data, predicting the risk of xerostomia for the greater population of HNC patients receiving radiotherapy. Therefore, it was not considered appropriate to exclude patients with baseline xerostomia. Baseline scores were also not available for all patients as this assessment has only recently been included in the registry. In future analyses, baseline xerostomia will be considered as a variable in the model. Notably, older age was highly significant in all models, likely as a surrogate for baseline xerostomia, as this is related to age in general and increased medication in particular (8).

Xerostomia is typically assessed by direct measurement of salivary-flow rate, by self-reported questionnaires or by scoring methods, as in the present study. Salivary-flow rates provide important information about salivary gland function and can be performed from each major gland or from a mixed sample of the oral fluids, often termed whole saliva. However, xerostomia is experienced differently by individuals (14) and is not identical to hyposalivation since it also depends on changes in the quality of saliva even with unchanged salivary-flow rate. Therefore patients may complain of dry mouth despite adequate salivation (27). In the current study, the oncologists perceived grade-2 xerostomia (moderately dry) to be less specific than grade 3 and 4 (completely dry and fibrosis, respectively). This may have contributed to the lower predictive power of the low-grade models; predictions made using the high-grade models are more reliable. However, since grade ≥2 xerostomia is problematic for the patients and relatively prevalent it is important to avoid also grade 2, if possible.

Another factor possibly contributing to the modest predictive power of the models is the variability in the time between radiotherapy and registered follow-up in the cohort. While focusing on a fixed time after treatment was not possible for our data set, this might have improved the performance of the model since there is a time dependence of incidence of xerostomia after treatment (15). As future work we plan to fit a model to a subset of the patients with follow-up data in a limited time interval. As well as revealing the importance of the follow-up time, this will also facilitate comparisons with studies using a fixed follow-up time. Future work will also explore the significance of HPV status for the risk of xerostomia after radiotherapy. Further, the predictive power may have been limited by the omission of HPV status, the patient-specific chemotherapy dose and alcohol consumption as explanatory variables; the two latter variables were not recorded in the registry and were not available for analysis.

IMRT has been shown to be associated with a lower incidence of severe xerostomia compared to 3D-conformal radiotherapy (14). Still, treatment with IMRT was not selected as an inclusion criterion in the current study since any relationship is expected to be indirect, through its impact on the mean parotid dose. Moreover, it was found that it would have made a very small difference to the model if non-IMRT patients had been excluded. Only age was not included in the XER_G≥2_ models; for all other variables, hazard ratios and *p*-values remained similar to those in the presented models. The value of the C statistic reflected a lower model performance when limiting the size of the cohort accordingly.

The *physical* mean parotid dose was used in this study, i.e., without correcting for fraction size per voxel or different fractionation schedules, since dose-volume histograms were not available for conversion to BED. The sensitivity of the results to this limitation was explored by converting the mean dose values to BED; this differs from the actual mean BED by around 1–15% (according to a comparison for a limited number of patients) since each dose bin was not converted before calculating the mean. It was seen, however, that while the predictive power of the models did not consistently improve they were somewhat sensitive to the representation of dose. Future work is planned to collect dose-volume histograms and refit the model with the dose variables in BED. Other sources of uncertainty in the dose variables are, as mentioned previously, that the small contribution from brachytherapy for 25% of the patients was ignored, but also the difference between planned and delivered dose. It has been shown that the actual mean dose to the parotids can increase by 10% or more compared to the planned dose, due to a gradual migration of the gland toward the high-dose volume over the course of treatment (28, 29). Furthermore, the identification of the parotid tissue in the CT images can be difficult, resulting in an uncertainty even in the planned dose.

The registry includes follow-up data for about 70% of the HNC patients treated with radiotherapy. However, since the missing data is mainly explained by a logistical failing in the data collection, patients were likely excluded without bias and thus the lack of full compliance should have a negligible impact on the results.

The fact that the current study included a large number of patients treated consecutively in our institution made it possible to develop a model more representative for the population it will be applied to, compared to models from controlled clinical trials. The diversity naturally occurring in the population is present in the sample and many relevant variables were candidates for inclusion in the models. By using Cox regression the time factor in the follow-up data was naturally accounted for and the risk of xerostomia can be predicted for different times after treatment. However, as a result of the inherent diversity in the dataset, it was found that the fitted model was very sensitive to small adjustments to the patients included in the analysis, despite the great size of the dataset. It was of particular interest to study the risk of xerostomia as a function of OAR dose, but these relationships were relatively weak, and in the case of grade ≥2 xerostomia an *inverse* dose-response relationship was found. The latter was unexpected but probably a result of indirect correlations or bias, which are more likely to appear in a dataset from a registry compared to a clinical trial. In line with our results, there are some indications that when patients have been treated with IMRT, resulting in lower parotid dose and steeper dose gradients compared to 3D-conformal radiotherapy, the strong association between xerostomia and mean parotid dose observed historically (22, 30) can be expected to be weaker or completely absent (31, 32). In the current analysis, the inverse dose-response found when forcing the dose variable into the multivariate models for XER_G≥2_ made the model unsuitable as a decision support tool and a nomogram was instead produced for a model which did not contain dose. Thus, unlike the nomogram for XER_G≥3_, this cannot be used to guide radiotherapy treatment planning but is nonetheless useful for managing the risk of moderate xerostomia.

Models developed based on real-world data are a valuable complement to controlled clinical trials and are particularly suitable as decision-support tools in a learning healthcare system (16). The analysis of registry data needs to be performed carefully, taking advantage of the heterogeneity of the population while selecting endpoints, variables and variable parameterization to obtain a useful model. For example, 2c was selected as reference for N stage since it was discovered that this disease stage was associated with a higher risk of xerostomia, even compared to stage 3 (although the prediction for stage 3 could be uncertain, given the few cases included in the dataset and that N3 was not statistically different from N2c as shown by the *p*-value in Supplementary Table A2). It was speculated that this is related to the bilateral location of involved lymph nodes with N2c. It is also important to not overinterpret the nomogram and assume that all aspects are statistically significant. For example, the model behind the nomogram in Figure 3 does not establish the relationship between N0 and N1, only each category’s difference from the reference category, N2c. There is no reason to suppose that N0 implies a higher risk compared to N1, and the small difference seen in the nomogram is unlikely to result in misleading predictions.

If the model is developed to improve future treatment plans it is important to consider possible mechanisms behind observed relationships, i.e., causality. In the current analysis it was felt that external validation would be required to confirm the lower risk associated with hyperfractionated/accelerated treatment schedules and induction chemotherapy, to rule out false correlations. The only support in the literature for the former is weak given the few patients studied and limited dose-volume information (33). It is hoped that future studies can confirm or dement the causality of these relationships.

## Conclusion

Late xerostomia after radiotherapy can be modeled with reasonable predictive power based on registry data, providing valuable alternatives to models developed on cohorts with stricter inclusion criteria. Similarities with similar models were observed but as the first Cox regression model for xerostomia, some important lessons were learned. The variables included and the performance of the model depend strongly on the grade of the endpoint, the patient selection and the candidate variables considered. The role of the parotid dose may be of lower importance compared to some clinical variables in a heterogeneous population. The results also suggest that an accelerated treatment schedule or induction chemotherapy may be associated with a lower risk of xerostomia, but until this has been confirmed a model excluding these variables can be used. The risk of modeling indirect relationships, given the unavoidably heterogeneous registry data, needs to be carefully considered in the interpretation of the results.

## Data Availability Statement

The datasets generated for this study will not be made publicly available. The patients participating in the study have not consented to their data being publicly available.

## Ethics Statement

The studies involving human participants were reviewed and approved by the Stockholm Regional Ethics Committee. The patients/participants provided their written informed consent to participate in this study.

## Author Contributions

EO, IL, and CM contributed to the conception and design of the study and interpreted the results together with EB and AE. AB was also involved in the design and interpretation of the statistical methodology and performed the analysis. CM, EB, AE, GA, SF, CL, and HS contributed to the data collection and IL to the registry design and management. GG and SF facilitated the development of the registry and advised on the scientific contents of the study. EO compiled and presented the results. The manuscript was written by EO, IL, and CM. All authors reviewed and approved the manuscript.

## Conflict of Interest

The authors declare that the research was conducted in the absence of any commercial or financial relationships that could be construed as a potential conflict of interest.
