# Supplementary material for: Modeling of Xerostomia After Radiotherapy for Head and Neck Cancer: A Registry Study
Source: Front Oncol. 2020 Aug 14;10:1647. doi: 10.3389/fonc.2020.01647 (PMC7456883; doi:10.3389/fonc.2020.01647)

Supplementary Material

# Univariate analysis

Table A1. Hazard ratios and corresponding p values (in brackets) for the Cox regression univariate analysis. The xerostomia endpoints were grade ≥2 (XER_G≥2_) and grade ≥3 (XER_G≥3_). Variables considered in the multivariate analysis are highlighted in bold. For variables with all values missing, one or more group contained too few events. The association between radiotherapy technique and xerostomia was not considered in the multivariate analysis, despite its statistical significance.

| **Endpoint** |  | **XER_G≥2,all_** | **XER_G≥3,all_** |
| --- | --- | --- | --- |
| D_tot_ |  | **0.995 (0.45)** | **1.02 (0.35)** |
| D_contra_ |  | **1.01 (0.065)** | **1.03 (0.13)** |
| D_ipsi_ |  | **0.991 (0.033)** | 1.003 (0.81) |
| Fractions per week;  reference = 5 |  |  |  |
| 6 |  | **1.2 (0.10)** | - |
| 10 |  | **0.28 (<0.01)** | - |
| Age |  | **1.01 (0.022)** | **1.06 (<0.01)** |
| Gender; reference = male |  |  |  |
| Female |  | 0.90 (0.32) | 0.79 (0.47) |
| Tumor location;  reference = oral cavity |  |  |  |
| Oropharynx |  | **1.10 (0.48)** | 1.75 (0.25) |
| Other |  | **0.66 (0.023)** | 0.86 (0.82) |
| T stage; reference = 4 |  |  |  |
| 0 |  | 0.77 (0.31) | 1.58 (0.55) |
| 1 |  | 0.86 (0.39) | 0.62 (0.50) |
| 2 |  | 0.98 (0.89) | 1.59 (0.40) |
| 3 |  | 1.11 (0.54) | 2.26 (0.17) |
| N stage; reference = 2c |  |  |  |
| 0 |  | **0.56 (<0.01)** | - |
| 1 |  | **0.49 (<0.01)** | - |
| 2a/b |  | **0.65 (<0.01)** | - |
| 3 |  | **0.32 (0.054)** | - |
| Smoking status;  reference = no/never |  |  |  |
| Smoker |  | **1.21 (0.19)** | **2.06 (0.12)** |
| Previous |  | **1.03 (0.78)** | **1.76 (0.15)** |
| Concomitant chemotherapy;  reference = no |  |  |  |
| Cisplatin |  | **1.69 (<0.01)** | **2.35 (0.020)** |
| Erbitux |  | **1.45 (<0.01)** | **0.77 (0.57)** |
| Induction chemotherapy;  reference = no |  |  |  |
| Yes |  | **0.59 (<0.01)** | 0.79 (0.50) |
| Radiotherapy technique; reference = 3D-CRT |  |  |  |
| IMRT |  | 3.32 (<0.01) | 4.70 (0.037) |
|  |  |  |  |

# Multivariate analysis without dose

Table A2. The model for XER_G≥2_ in Table 3 is not suitable as a decision support tool since a higher D_tot_ appears to have a protective effect for the endpoint. The nomogram is therefore presented for an alternative model where D_tot_ was not forced to be included. Since D_tot_ had a high p value in the univariate analysis it did not get selected into the multivariate model without this criterium. The model behind the nomogram in Figure 1 is listed below.

| **Included variables** |  | **Hazard ratio (p value)** |
| --- | --- | --- |
| Tumor location; reference = oral cavity |  |  |
| Oropharynx |  | 0.99 (0.96) |
| Other |  | 0.56 (<0.01) |
| N stage; reference = 2c |  |  |
| 0 |  | 0.64 (0.02) |
| 1 |  | 0.59 (0.018) |
| 2a/b |  | 0.70 (0.034) |
| 3 |  | 0.32 (0.056) |
| Concomitant chemotherapy; reference = no |  |  |
| Cisplatin |  | 1.82 (<0.01) |
| Erbitux |  | 1.31 (0.06) |


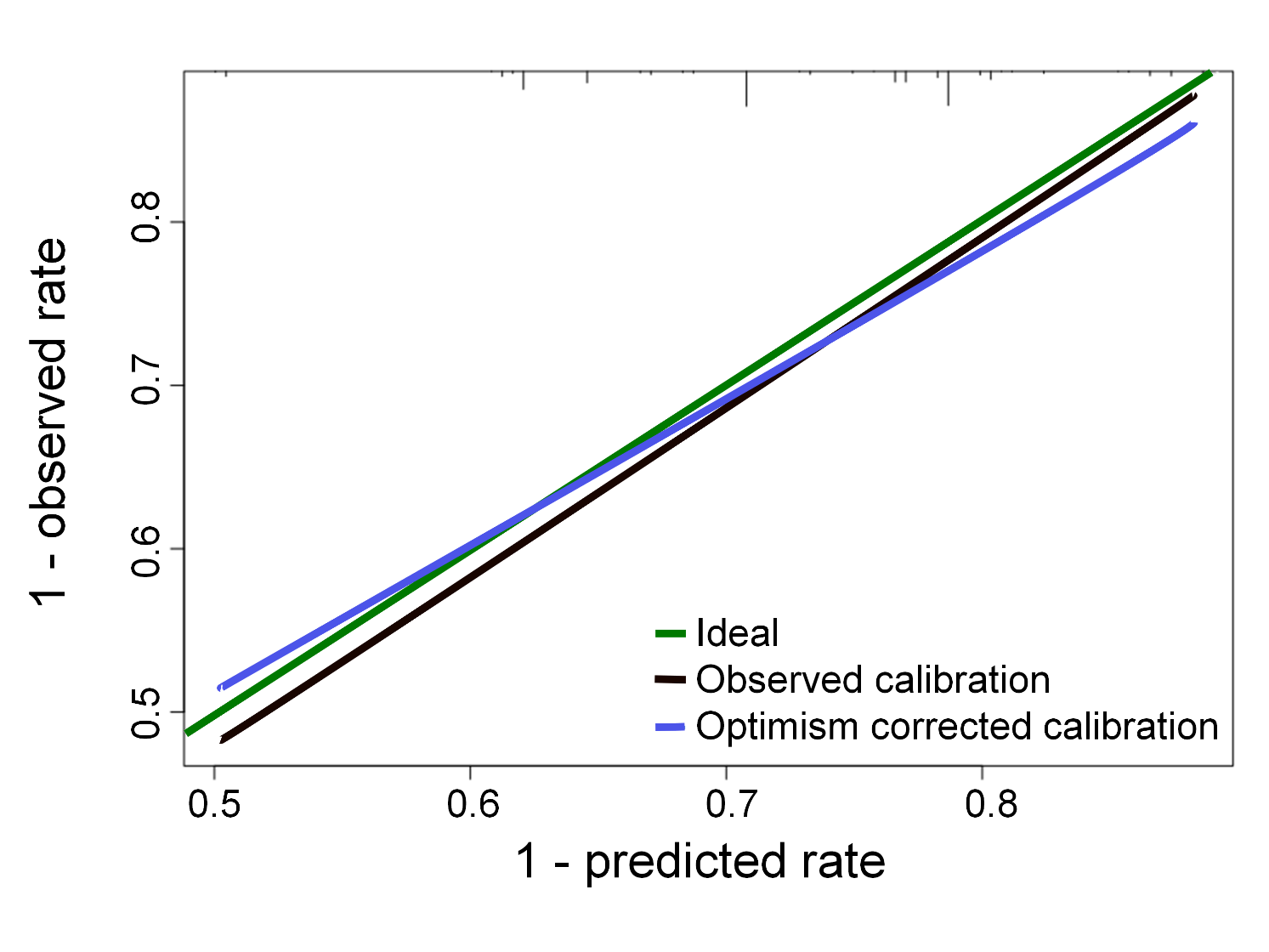


Figure A1. Calibration plot for the model in Table A2. The histogram on the upper x axis represents the frequency distribution of 1 minus the predicted probabilities (c.f. a survival analysis).

# Nomogram example

The nomogram below illustrates how to read the nomograms. For an example patient at an age of 80 years, who does not receive concomitant chemotherapy and who receives a mean dose to the total parotid volume of 20 Gy, the total points is the sum of the points indicated by each variable respectively, i.e. 82 + 30 + 52 = 164. The total points then indicates the risk of grade ≥3 xerostomia at each listed time point, below: 4.0% at 9 months, 4.5% at 12 months and 14% at 24 months after radiotherapy.


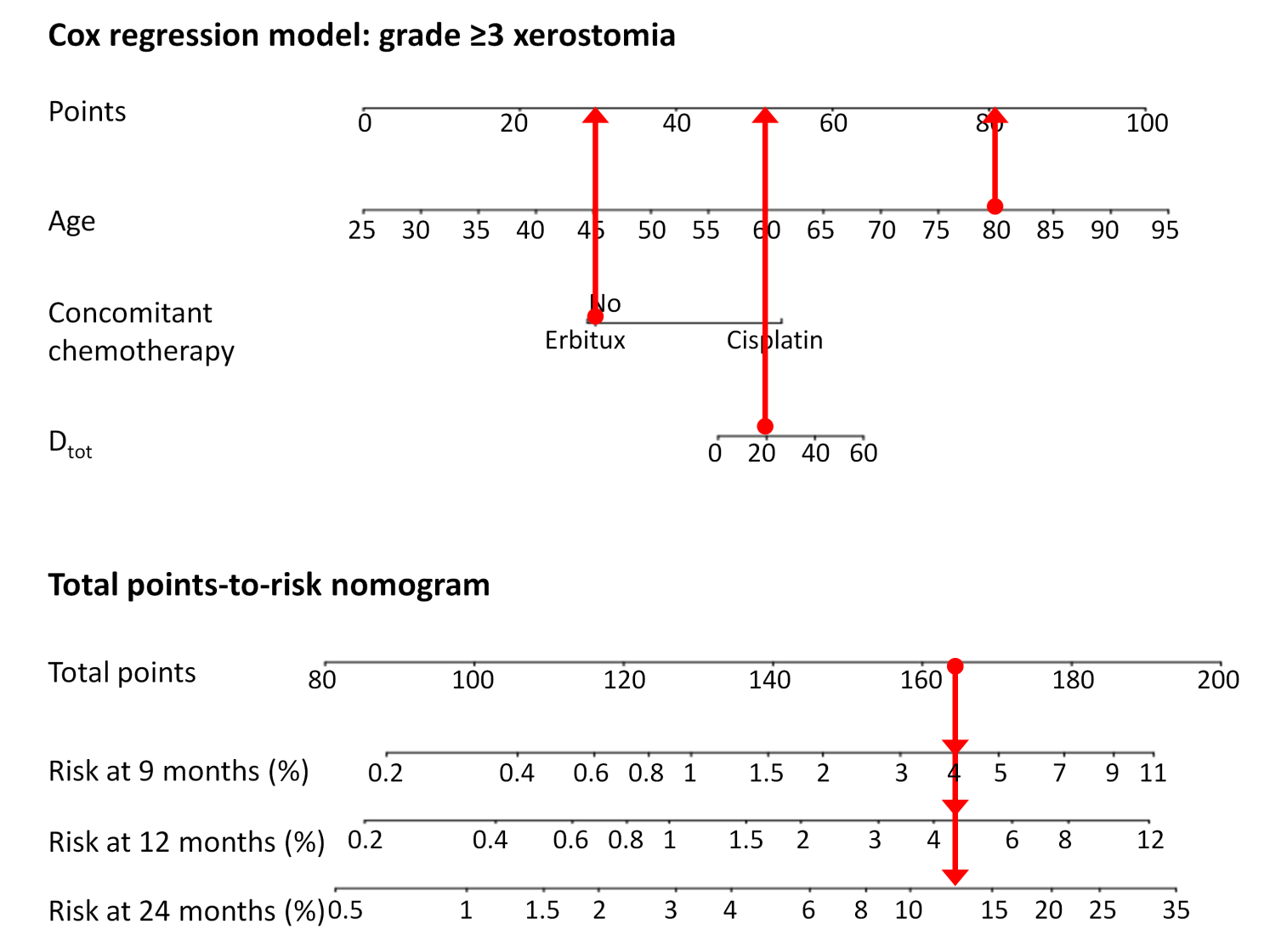

Supplement: Supplementary file 1 [file Data_Sheet_1.docx]
